# The Predictors and Outcomes of Functional Mitral Stenosis following Surgical Mitral Valve Repair: A Retrospective Analysis

**DOI:** 10.3390/jcdd10110470

**Published:** 2023-11-19

**Authors:** Yu-Ning Hu, Wen-Huang Lee, Meng-Ta Tsai, Yi-Chen Wang, Chao-Jung Shih, Yu-Ching Huang, Jun-Neng Roan

**Affiliations:** 1 Division of Cardiovascular Surgery, Department of Surgery, National Cheng Kung University Hospital, College of Medicine, National Cheng Kung University, Tainan 704, Taiwan; windermere0209@gmail.com (Y.-N.H.); dongsar@gmail.com (M.-T.T.); wyckate@hotmail.com (Y.-C.W.); juliecvsnckuh@gmail.com (C.-J.S.); jadeching4@gmail.com (Y.-C.H.); 2 Division of Cardiology, Department of Internal Medicine, National Cheng Kung University Hospital, College of Medicine, National Cheng Kung University, Tainan 704, Taiwan; wenhuanglee@gmail.com

**Keywords:** mitral valve regurgitation, mitral valve repair, functional mitral stenosis

## Abstract

To optimize mitral valve repair outcomes, it is crucial to comprehend the predictors of functional mitral valve stenosis (FMS), to enhance preoperative assessments, and to adapt intraoperative treatment strategies. This study aimed to identify FMS risk factors, contributing valuable insights for refining surgical techniques. Among 228 selected patients, 215 underwent postoperative echocardiography follow-ups, and 36 met the FMS criteria based on a mean trans-mitral pressure gradient of >5 mmHg. Patients with FMS exhibited higher pulmonary systolic arterial pressure and increased late mortality during the follow-up. Univariable logistic regression analysis identified several risk factors for FMS, including end-stage renal disease, anterior leaflet lesion, concomitant aortic valve replacement, smaller ring size, ring type, and neochordae implantation. Conversely, resection alone and resection combined with neochordae implantation had protective effects against FMS. Multivariable logistic regression analysis revealed that smaller ring sizes and patch repair independently predicted FMS. When focusing on degenerative mitral regurgitation, the neochordae implantation without resection in leaflet repair, emerged as an independent predictor of FMS. Surgeons should weigh the substantial impact of surgical procedures on postoperative trans-mitral pressure gradients, emphasizing preoperative evaluation and techniques such as precise ring size assessment and effective leaflet management.

## 1. Introduction

In the surgical management of most types of mitral insufficiency, including degenerative mitral insufficiency, functional mitral insufficiency, and infective mitral insufficiency, mitral valve (MV) repair has been established as a more effective approach than mitral valve replacement [1,2]. The benefits of MV repair include lower surgical risks, improved long-term survival, and fewer valve-related complications, which have been demonstrated across all population groups, including older adults [3]. The American College of Cardiology/American Heart Association guideline [4] recommends MV repair as the standard treatment for primary mitral regurgitation (MR), classifying it as a class I recommendation.

Among the techniques used in MV repair, ring annuloplasty has been proposed as a method to reshape the mitral annulus and reduce MR recurrence [5]. However, a consensus has not yet been reached regarding the best choice of annuloplasty, specifically between complete rings and partial bands, and the selection of semi-rigid or rigid materials [6,7,8,9]. Some surgeons even opt for biodegradable rings [10]. Although using a complete ring has been associated with a lower MR recurrence rate than a partial band or no annuloplasty at all [11,12,13], it also results in a higher incidence of functional mitral stenosis (FMS) [14,15].

FMS, primarily caused by restrictive annuloplasty, is associated with inferior exercise tolerance, failed left atrial remodeling, new-onset atrial fibrillation (Af), elevated pulmonary artery pressure, residual tricuspid regurgitation (TR), and poor long-term prognosis [16,17,18,19,20].

FMS is defined as a resting mean trans-mitral pressure gradient (TMPG) of >5 mmHg or MV area of <1.5 cm^2^ at rest [20,21], and a mean TMPG of >15 mmHg and pulmonary artery systolic pressure of >60 mmHg during exercise [22]. Previous studies [23,24] have indicated that the use of rigid or undersized rings may increase the risk of a smaller subsequent MV area, leading to FMS.

In this tertiary medical center, the annuloplasty rings used for all MV repairs are semi-rigid, complete annuloplasty rings, including the Edwards Physio I, Edwards Physio II, Edwards IMR ring (Carpentier–McCarthy–Adams IMR ETlogix annuloplasty ring), and LivaNova Memo 3D ring. This study aimed to retrospectively analyze patients who underwent MV repair with annuloplasty at this institution over 6 years. The study focuses on assessing the preoperative baseline characteristics, leaflet management procedure, and choice of ring type and size during the operation. Furthermore, the study evaluated preoperative and postoperative echocardiographic data to identify patients meeting the definition of FMS. This study also tried to identify the predictors and outcomes of FMS to provide information for future operative procedures and ring selection, thereby improving patients’ long-term outcomes and quality of life.

## 2. Materials and Methods

This retrospective review study included consecutive patients who underwent MV repair with annuloplasty at National Cheng Kung University Hospital between January 2015 and July 2022. The institution IRB has approved the study (B-ER-111-274). The study included elective, urgent, and emergency surgeries for a range of mitral pathologies, encompassing primary MR, which includes degenerative and endocarditis-related causes, as well as secondary MR, including functional and ischemic mitral diseases. Redo operations, including re-repair procedures, were also included in the analysis. However, patients who received leaflet repair without annuloplasty were excluded. Additionally, patients diagnosed with rheumatic mitral regurgitation were also excluded from the analysis. Rheumatic mitral disease often presents concomitant MR and mitral stenosis. The presence of leaflet calcification and thickening before surgery is characteristic of this condition [25,26,27]. This pre-existing condition could potentially lead to residual mitral stenosis following mitral valve repair, thereby potentially affecting the accurate diagnosis of FMS.

Various surgical variables were documented during the intraoperative phase, including concomitant procedures, size of the mitral ring annuloplasty, choice of mitral ring type, and the specific approaches taken to leaflet management. The leaflet repair procedures were comprehensively documented using the following classifications: resection only, involving either triangular resection or quadrangular resection; resection combined with neochordae implantation; neochordae implantation without accompanying resection; and chordae transfer. All neochordae implantations were performed utilizing CV-4 (GORE-TEX^®^ Suture, WL Gore & Associates, Newark, DE, USA), whereas chordae transfer procedures exclusively involved posterior major chordae transfer to the anterior leaflet. These four distinct documentation categories were utilized. Patients presenting with leaflet perforation or restriction necessitating patch repair or augmentation were documented under the patch repair classification. Edge-to-edge repair procedures, whether performed as the sole leaflet repair approach or in combination with other leaflet repair techniques, were documented under the edge-to-edge repair category. Notably, both patch repair and edge-to-edge repair techniques may be employed in conjunction with other leaflet procedures. Patients who underwent only annuloplasty were not categorized in any specific leaflet management classification. In our institution, under typical circumstances, all mitral valve repairs are generally accompanied by ring annuloplasty, except in cases where extensive mitral annulus calcification precludes ring annuloplasty, or when the patient is a child. However, for the purposes of this study, we only included patients who underwent ring annuloplasty.

Different pathologies affecting MV necessitate distinct repair strategies. In cases of degenerative MR, surgeons commonly employ resection with or without neochordae implantation. Neochordae implantation without leaflet resection is also selected when there is minimal leaflet prolapse and/or an anterior leaflet lesions. Chordae transfer may be considered for certain patients if their subvalvular apparatus is suitable. In contrast, infective endocarditis-related MR calls for a more comprehensive approach; surgeons must completely resect the infectious tissue and subsequently evaluate whether patch repairs with or without neochordae are needed to reconstruct the MV.

Secondary MR is addressed through either true-sized or downsized annuloplasty, which serves to restore proper MV function and mitigate regurgitation. In cases where the surgeon determines that a simple annuloplasty may not yield optimal repair results due to severe left ventricular dilatation, interventions, such as chordal translocation or surgical edge-to-edge repair, are employed.

The selection of the leaflet management technique is guided by intraoperative assessment and the preference of the surgeon.

Following MV repair, anesthesiologists conduct transesophageal echocardiography to ensure completion of the repair before proceeding to close the sternum. Both 2D and 3D images are utilized to assess the morphology and mobility of the MV. Color Doppler imaging is employed to detect any residual MR or the presence of MV stenosis (MS). The transvalvular mean pressure gradient is measured to evaluate the possibility of MS in the patients showing diastolic turbulence across the MV. The threshold for defining mild or lower MS is a TMPG ≤ 5 mmHg. If both MR and MS are classified as mild or lower, the sternum is closed, indicating an acceptable repair. Conversely, patients presenting with more than mild MR or MS undergo re-crossclamping and may require additional repair or valve replacement.

If differences in opinion regarding MV evaluation arise between cardiac surgeons and anesthesiologists, an echocardiologist is consulted to comprehensively evaluate the MV in the operating room.

In this study, the patients were retrospectively divided into a FMS group, which comprised patients with a mean TMPG > 5 mmHg, and a no FMS (nFMS) group, which included those with a mean TMPG ≤ 5 mmHg. The primary outcome measure was long-term all-cause mortality, and secondary outcomes included cardiovascular event-related late death [28], moderate and severe MR recurrence, pulmonary artery systolic pressure (PASP), and left atrial (LA) size remodeling.

FMS predictors were assessed based on patients’ preoperative baseline characteristics and intraoperative surgical management variables that demonstrated significant differences between the FMS and nFMS groups.

### Statistical Analysis

Statistical analysis was performed using appropriate methods to compare variables and evaluate predictors and outcomes in the FMS and nFMS groups. Continuous variables were expressed as means and standard deviations, whereas categorical variables were presented as frequencies and proportions. To compare preoperative characteristics, intraoperative characteristics, and outcomes between the FMS and nFMS groups, a two-sample Student’s *t*-test was employed for continuous variables, and a Chi-square test or Fisher’s exact test was used for categorical variables. We utilized Kaplan–Meier survival curves to analyze long-term survival rates, and the Cox regression analysis was employed to assess the long-term echocardiography outcomes. The evaluation of preoperative and postoperative LA size remodeling was conducted using paired *t*-tests. FMS-associated predictors that exhibited a *p*-value < 0.1 in the two-group comparison were included in the univariable logistic regression analysis. Subsequently, predictors with a *p*-value < 0.1 in the univariable analysis were incorporated into the multivariable logistic regression analysis. The variance inflation factor was calculated during multiple regression analyses to assess multicollinearity. The significance level was set at *p* < 0.05. All statistical analyses were performed using MedCalc Software (MedCalc^®^ Statistical Software version 22.007, MedCalc Software Ltd., Ostend, Belgium; https://www.medcalc.org; accessed on 31 August 2023).

## 3. Results

**Figure 1:**
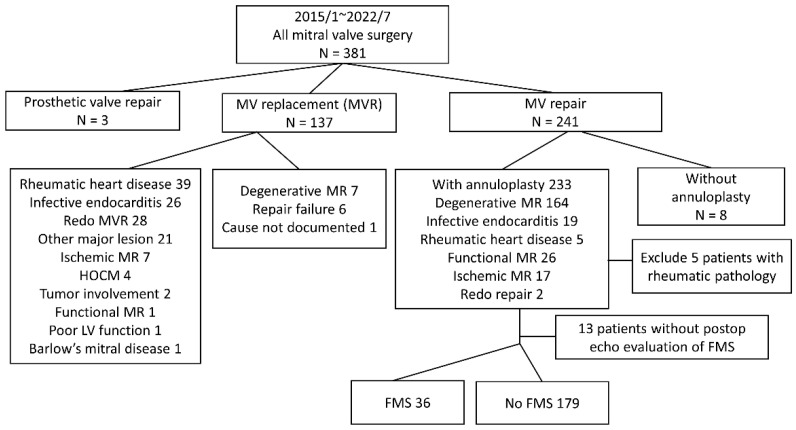
Flowchart of case enrollment. FMS, functional mitral stenosis; HOCM, hypertrophic obstructive cardiomyopathy; MR, mitral regurgitation; MV, mitral valve; MVR, mitral valve replacement.

During the study period, a total of 241 consecutive patients underwent MV repair at this institution (Figure 1). Five patients with rheumatic MR and eight patients who underwent mitral valve repair without annuloplasty were excluded in the analysis, leaving 228 patients for further analysis. Most MR cases were attributed to degenerative causes and 19 cases were associated with infective endocarditis. Secondary MR, encompassing functional and ischemic etiologies, was identified in 43 patients. Among these, 215 patients had at least one follow-up echocardiography, of which 36 were classified as having FMS based on a mean TMPG > 5 mmHg. Table 1 presents the baseline characteristics of the FMS and nFMS groups. No significant differences were observed between the two groups in terms of age, sex, body surface area (BSA), and preoperative echocardiographic findings, including left ventricular ejection fraction (LVEF), an incidence of LVEF < 40%, PASP, PASP ≥ 60 mmHg, severe TR, left ventricular end-diastolic diameter (LVEDD), left ventricular end-systolic diameter (LVESD), annulus dilatation, and LA diameter. Likewise, MR pathologies, whether primary or secondary MR, showed no statistically significant differences between the FMS and nFMS groups (*p* = 0.121). Annulus dilatation is recognized when the annulus-to-leaflet ratio exceeds 1.3 or when the diameter surpasses 35 mm [29] in the parasternal long-axis transthoracic echocardiography view, of which no significant difference between the two groups was shown. The extent of segment involvement, which indicates the number of affected segments within the six mitral parts (A1, A2, A3, P1, P2, P3), serves as an indicator of the complexity of the MV repair. This measure also revealed no significant difference between the two groups. However, the location of the leaflet lesion significantly influenced FMS occurrence (*p* = 0.046).

Table 2 presents the operative characteristics. In the context of concomitant procedures, concomitant aortic valve replacement (AVR) exhibited a significantly higher incidence of FMS. However, other concomitant procedures, including coronary artery bypass grafting (CABG), Maze procedure, tricuspid valve surgery, and aorta procedure did not significantly influence the occurrence of FMS. Regarding mitral ring size, in the FMS group, the range of used ring sizes spanned from 26 mm as the smallest to 32 mm as the largest, with a median of 28 mm. In contrast, within the nFMS group, the range of used ring sizes extended from 24 mm as the smallest to 38 mm as the largest, with a median of 30 mm. For a detailed distribution of mitral ring sizes, we present Supplementary Figure S1. Predictably, a smaller mitral ring size was associated with a higher incidence of FMS (*p* = 0.0004). Furthermore, there was a significant difference in mitral ring type between the FMS and nFMS groups (*p* = 0.019). Of the leaflet management techniques, those involving patch repair and neochordae-only in leaflet repair were also associated with a higher incidence of FMS. Conversely, repair with resection only and resection combined with neochordae showed a lower FMS incidence.

**Figure 2:**
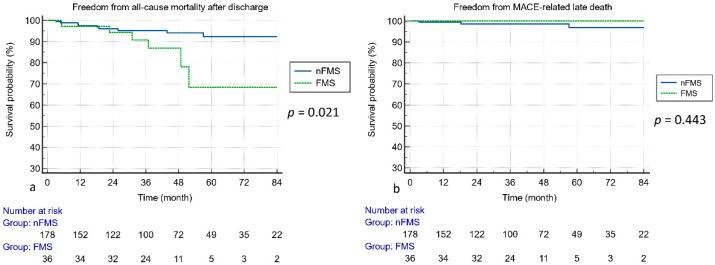
Kaplan–Meier curve in the FMS and nFMS groups. (**a**) Freedom from all-cause mortality after the initial survival to discharge. (**b**) Freedom from cardiovascular event-related late death.

**Figure 3:**
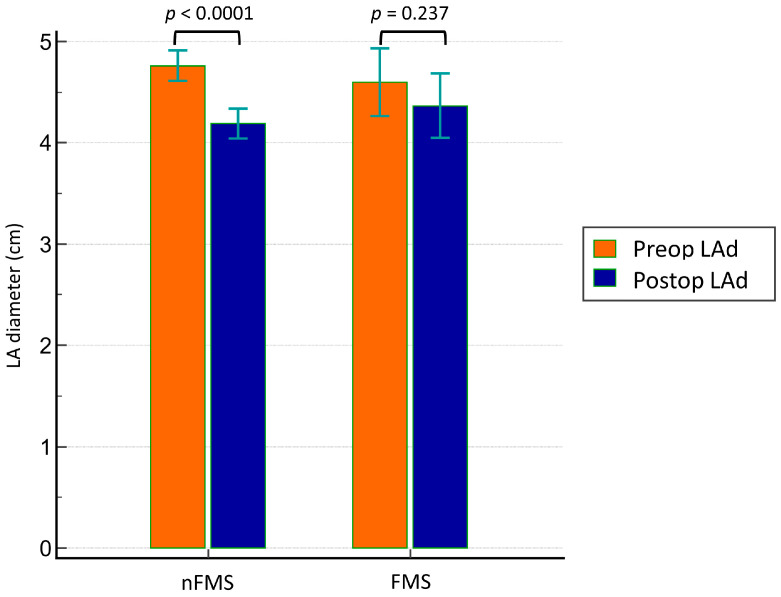
Preoperative and postoperative LA diameters in the FMS and nFMS groups. Preop, preoperative; Postop, postoperative; LAd, left atrial diameter.

Table 3 and Figure 2 present the primary and secondary outcomes. Although no significant differences in surgical mortality and hospital mortality were observed, there was an indication of a higher probability of late death in the FMS group (Figure 2a, *p* = 0.021). No significant differences in cardiovascular event-related late death were found between the two groups (Figure 2b, *p* = 0.443). The FMS group exhibited higher postoperative PASP, mean TMPG, and peak TMPG values. However, no significant differences in the MV area and postoperative TR grading were noted between the two groups. Regarding LA size remodeling, although both the preoperative and postoperative LA diameters were not significantly different between the FMS and nFMS groups, paired *t*-tests revealed poorer LA size remodeling in the FMS group than in the nFMS group (Figure 3, *p* = 0.237 in the FMS group vs. *p* < 0.0001 in the nFMS group).

**Figure 4:**
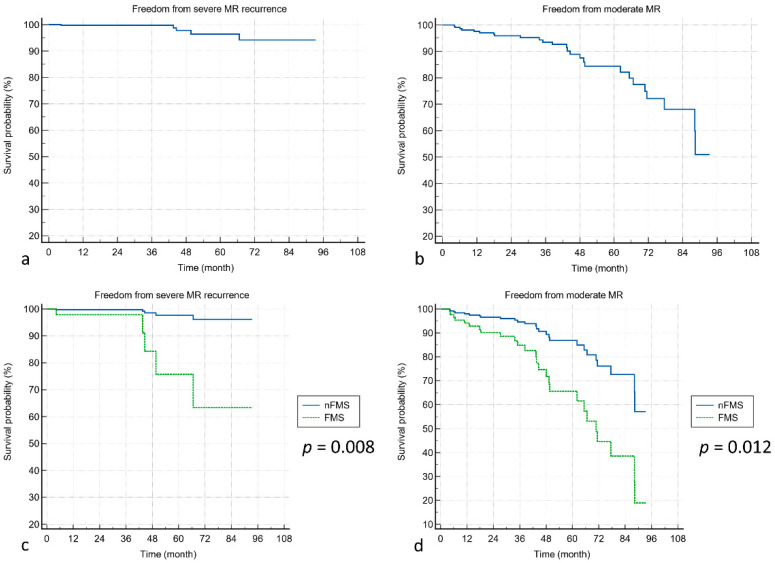
Mitral regurgitation recurrence following mitral valve repair. (**a**) Freedom from severe MR recurrence in all patients. (**b**) Freedom from moderate MR recurrence in all patients. (**c**) Freedom from severe MR recurrence between the FMS and nFMS groups. (**d**) Freedom from moderate MR recurrence between the FMS and nFMS groups.

In patients with a postoperative echocardiography follow-up, the 1-, 3-, and 5-year freedom rates from severe MR recurrence were 99.7%, 99.7%, and 96.4% (Figure 4a), whereas the 1-, 3-, and 5-year freedom rates from moderate MR recurrence were 97.6%, 93.5%, and 84.3%, respectively (Figure 4b). The FMS group showed a significantly higher incidence of MR recurrence, either in severe (Figure 4c, *p* = 0.008) or moderate grading (Figure 4d, *p* = 0.012).

To account for the potential confounding effect of residual MR on the TMPG, 26 patients with more than mild residual MR were excluded from the FMS predictor analysis. Among the remaining 189 patients with mild or lower MR, 28 patients had a resting mean TMPG > 5 mmHg. Predictors with a *p*-value < 0.1 in Table 1 and Table 2 were included in the univariable logistic regression analysis (Table 4). The analysis unveiled that ESRD, anterior leaflet lesions, concomitant AVR, smaller mitral ring sizes, ring types, and exclusively employing neochordae in leaflet management were identified as dependent factors predictive of FMS. In contrast, both resection alone and the combination of resection with neochordae implantation demonstrated protective effects against FMS. Multivariable logistic regression analysis indicated that the mitral ring size (odds ratio (OR): 0.691, 95% confidence interval (CI): 0.513–0.930, *p* = 0.014) and patch repair in leaflet management (OR: 92.905, 95% CI: 3.271–2638.442, *p* = 0.007) were independent FMS predictors. To assess the potential presence of multicollinearity, variance inflation factors were calculated during the multiple regression analysis. As shown in Table 4, all variance inflation factors for the predictors included in the analysis were < 3, indicating that multicollinearity is not a major concern.

### Subgroup Analysis

Given the potential complexity associated with encompassing all MR pathologies, which could introduce confounding factors when assessing the contribution of intraoperative repair techniques to FMS, we conducted a distinct analysis involving a patient subgroup exclusively composed of individuals with degenerative MR, totaling 161 cases.

In terms of baseline characteristics and preoperative data, there were significant differences between FMS and nFMS only in terms of age and the location of the leaflet lesion (Table 5).

Concerning intraoperative characteristics, the factors encompassing AVR, mitral ring size, mitral ring type and leaflet repair techniques—specifically neochordae implantation alone, edge-to-edge repair, and combined resection with neochordae implantation—displayed significant differences between the FMS and nFMS groups, as indicated in Table 6.

When it comes to surgical outcomes, the FMS group exhibited higher values for PASP, mean TMPG, and peak TMPG compared with the nFMS group, with no statistically significant differences observed in other parameters (Table 7).

Table 8 presents the outcomes of a logistic regression analysis aimed at identifying predictors of FMS within the degenerative MR group. The univariable analysis revealed that anterior leaflet lesion, mitral ring size, mitral ring type, and neochordae implantation only in leaflet repair were dependent predictors of FMS. The results of the multivariable analysis demonstrated that neochordae implantation alone in leaflet management was an independent predictor of FMS. Furthermore, it iss worth mentioning that all the variance inflation factors for the predictors in the analysis of degenerative MR were also less than three.

## 4. Discussion

After MV repair with annuloplasty, FMS occurred in 16.7% of all non-rheumatic mitral pathologies. Patients with FMS experienced inferior long-term overall survival, although no significant difference in cardiovascular event-related late death was observed when compared with the nFMS group. The FMS group exhibited significantly higher PASP values and poorer LA size remodeling. Furthermore, patients with a resting mean TMPG > 5 mmHg had higher MR recurrence rates following MV repair, in both severe and moderate grading. Regarding the prediction of FMS, the results of univariable logistic regression identified several factors as dependent predictors, which included ESRD, anterior leaflet lesions, concomitant AVR, smaller ring size, mitral ring type, and the exclusive use of neochordae in leaflet repair. Conversely, resection alone and the combination of resection with neochordae implantation demonstrated protective effects against functional mitral stenosis. Subsequent multivariable logistic regression analysis confirmed that smaller ring sizes and patch repair in leaflet management emerged as independent predictors of FMS. In a subgroup analysis specifically targeting patients with degenerative MR, the univariable analysis indicated anterior leaflet lesions, mitral ring size, mitral ring type, and sole neochordae implantation in leaflet repair as factors associated with FMS. However, in the multivariable analysis, only neochordae implantation without resection emerged as an independent predictor of FMS.

This study revealed that patients with FMS had inferior long-term overall survival to those in the nFMS group, although cardiovascular event-related late death did not differ significantly between the two groups. Kawamoto et al. [16] reported that FMS did not carry inferior long-term survival but caused an elevation in the pulmonary artery pressure, residual TR grade, and new-onset Af. Another study [18] also yielded comparable findings, indicating that FMS is linked to unfavorable intracardiac hemodynamics, higher B-type natriuretic peptide levels, reduced exercise capacity, and poorer quality of life. Kim et al. [30] reported that FMS increased the risk for new-onset atrial fibrillation, MV reoperation, and decreased long-term survival. In the present study, survival in the FMS group deteriorated after 2 years postoperatively, with most causes of death unrelated to cardiac adverse events.

In our study, patients did not require MV reinterventions, specifically due to FMS. MV reinterventions for severe FMS pose significant challenges as redo surgery possesses a relatively high risk and can be technically demanding. Transcatheter MV interventions, such as transcatheter edge-to-edge repair and transcatheter annuloplasty, are generally not suitable for patients with FMS. The outcomes of transcatheter MV implantation within a ring show suboptimal results compared with transcatheter MV implantation within the prosthetic valve [31]. Therefore, surgeons should focus on proactive measures to prevent FMS occurrence during surgical MV repair.

As our study was a retrospective cohort study, FMS was classified based on resting echocardiography evaluations with a mean TMPG > 5 mmHg. The presence of significant residual or recurrent MR can lead to an increase in LA volume and trans-mitral flow [24], consequently resulting in elevated TMPG. In order to consider the potential impact of residual or recurrent MR on TMPG, a total of 26 patients with more than mild MR were excluded from the predictor analysis.

The selection of different types of MV rings, such as the Edwards Physio I, Edwards Physio II, Edwards IMR ring, or LivaNova Memo 3D ring, demonstrated a significant difference between the two groups. Furthermore, the logistic univariable analysis revealed that the LivaNova Memo 3D ring had a higher incidence of FMS compared to the Edwards Physio I ring. Each distinct valve ring design, encompassing factors such as anteroposterior diameter, ring orifice area, and flexibility, possesses its own unique attributes. It is important to note that having the same ring size does not necessarily equate to having the same ring orifice area. Even when there is a consistent ring orifice area in vitro, variation in ring flexibility [32,33] could lead to different outcomes in patients. The selection of a specific type of mitral ring, with the exception of cases involving particular mitral regurgitation pathologies (such as the use of an IMR ring for ischemic MR), is frequently influenced by the surgeon’s individual experience and preferences.

Multivariable logistic regression analysis confirmed that a smaller ring size and patch repair were independent predictors of FMS. Consistent with the findings of most prior studies [14,15,23,24,34] investigating FMS following mitral valve repair, our study also demonstrates that annuloplasty with smaller ring sizes significantly elevates the risk of FMS. Thus, it is unsurprising that a reduced mitral ring size is associated with an increased FMS risk.

Regarding patch repair, only five patients underwent this procedure throughout the entire study, and all five patients had infective endocarditis. At our institution, when patch repair is necessary, we routinely utilize the Edwards bovine pericardial patch, which is treated with glutaraldehyde. This type of patch has previously been associated with instances of late calcification and stenosis [35,36]. We speculate that the use of glutaraldehyde-treated bovine pericardial patch could potentially lead to subsequent calcification of the patch, thereby contributing to the higher incidence of FMS following patch repair. Due to the extremely limited sample size and the use of a single type of patch material with a historically unfavorable long-term outcome, we believe that considering patch repair as an independent factor may be overly conclusive and likely not applicable to other studies.

To further evaluate whether intraoperative surgical procedures indeed affect the incidence of FMS, we conducted a subgroup analysis on patients with degenerative MR. The univariable analysis of degenerative MR showed that anterior leaflet lesions, mitral ring size, mitral ring type, and neochordae implantation only in leaflet repair were identified as dependent predictors of FMS. Furthermore, in the multivariable analysis, only neochordae implantation without resection emerged as an independent predictor of FMS. Interestingly, in the degenerative MR group, it is noteworthy that mitral ring size did not emerge as an independent predictor of FMS.

Regarding leaflet management strategies, Hibino et al. [24] reported that resection repair with small annuloplasty poses a higher FMS risk. However, a multicenter randomized controlled trial [37] demonstrated that mitral prolapse repair through either leaflet resection or preservation was associated with a similar TMPG. Another study [38] showed that both leaflet resection and chordal replacement repair techniques were effective in terms of TMPG, MV competence, and postoperative left ventricular function. Surprisingly, the current study showed the leaflet repair strategy that involved neochordae implantation without resection was associated with a significantly higher FMS incidence in the degenerative MR group.

This study has yielded unexpected outcomes that are not comparable with previous findings. To explore the potential presence of high multicollinearity among the predictors, we calculated the variance inflation factor during multiple regression analysis. The findings revealed that in both the overall non-rheumatic MR patient group and the specific degenerative MR subgroup, all variance inflation factors for the predictors examined in the multivariable analysis remained below three. This suggests that there is no significant issue of multicollinearity among the predictors.

In our study, the neochordae implantation without leaflet resection, also known as the respect strategy, resulted in higher TMPG values in the degenerative MR group. These findings diverge from those reported in previous studies, and a comprehensive explanation based solely on procedural factors remains elusive. We believe that certain shortcomings in our repair procedure might have contributed to the unpredictability of the results. Typically, surgeons opt for neochordae implantation without resection when the leaflet prolapse is deemed insignificant or when the lesion is located in the anterior leaflet. Insignificant leaflet prolapse implies that severe MR may be in its early stages, characterized by a relatively smaller left ventricle and left atrium, thereby posing challenges during repair. Additionally, the repair technique in the anterior leaflet is generally considered more challenging than that in the posterior leaflet.

Furthermore, instances of unsatisfactory leaflet repair could introduce complexity into the intraoperative assessment by surgeons, potentially influencing their decision to opt for downsized annuloplasty in an effort to reduce residual MR. It is crucial to highlight that an undersized ring may also result in elevated TMPG, thereby contributing to the development of FMS. As a result, we attribute the increased incidence of FMS in patients with degenerative MR who exclusively underwent neochordae implantation during leaflet repair to inappropriate ring sizing and/or inadequate management of leaflet tissue, rather than the neochordae implantation procedure itself. In terms of anatomical structure and pathophysiology, neochordae implantation does not inherently induce FMS. In this study, patients who underwent both leaflet resection and neochordae implantation had a lower probability of developing FMS. This observation is consistent with the earlier mentioned concept that neochordae implantation itself does not increase the likelihood of FMS occurrence. The key factor lies in leaflet management and ring sizing. Nevertheless, it is worth noting that both ring size and neochordae implantation alone did not exhibit a significant impact on the likelihood of moderate mitral regurgitation recurrence (ring size, OR 1.08, 95% CI 0.93–1.26, *p* = 0.299; neochordae implantation alone, OR 1.91, 95% CI 0.86–4.39, *p* = 0.124).

FMS is a genuine anatomical problem originating from the time of surgery [39], and surgeons must exercise caution, respecting the mitral leaflet and resecting it when necessary [40]. Excessive reliance on leaflet-sparing strategies without resection in degenerative MR repair may lead to unsatisfactory long-term outcomes. To the best of our knowledge, no previous studies have reported that a respect leaflet repair strategy can lead to a higher incidence of FMS.

### Limitations

This study has several limitations that should be considered when interpreting the results. First, the retrospective nature of the cohort design introduces potential biases related to the procedure details, including leaflet management, choice of ring type, and ring size, as these decisions were based on individual surgeons’ experience and preferences. This selection bias could have influenced the study outcomes. Second, the study’s sample size is relatively limited, which prevented the feasibility of conducting propensity score matching to address potential confounders adequately. This limitation may have affected the generalizability of the findings and the ability to control for all relevant variables. Third, the study encompassed patients with different types of MR; however, there was a significant disparity in the number of cases for each pathology. Different mitral pathologies, such as functional or ischemic MR, downsized, restrictive annuloplasty techniques may have been employed, potentially leading to a higher FMS incidence. The differences in MR pathologies may have had an impact on postoperative TMPG, consequently influencing the categorization of patients into those with FMS and those without. Fourth, it is important to note that the definition of FMS in this study solely relied on the resting mean TMPG without consideration of stress or exercise echocardiography. Additionally, the MV area was estimated by dividing 220 by the pressure half-time. As this is a retrospective study, routine 3D echo-imaging was not performed for all patients to calculate the MV area. Evaluating FMS based solely on resting echocardiography with TMPG may introduce a degree of grouping bias. Moreover, it is crucial to acknowledge that the current study lacks documentation of preoperative left ventricular diastolic dysfunction, a factor that could potentially offer insights into the extent of the trans-mitral gradient. Finally, the study involved seven cardiac surgeons performing MV repairs during the study period, with a significant proportion of procedures performed by a single surgeon. The varying levels of experience in MV evaluation, mitral annuloplasty, leaflet management procedures, neochordae implantation, and ring type selection among the surgeons may have introduced additional bias into the repair outcomes. Furthermore, for patients presenting with Type I or Type IIIb MR, downsized annuloplasty was performed. However, accurately quantifying the extent of the “downsize” for each patient can be challenging due to intersurgeon variations.

## 5. Conclusions

In this study, the predictive factors associated with FMS following MV repair include smaller ring sizes and patch repair in leaflet management. These findings suggest that the surgical procedure itself significantly influences postoperative TMPG. The expertise of surgeons in selecting the appropriate annular size and avoiding un-dersizing of the annuloplasty ring is fundamental in preventing the FMS. Surgeons should exercise caution and adopt a respectful approach toward the mitral leaflet, considering resection when deemed necessary, and remain vigilant about the potential occurrence of FMS, particularly during ring size evaluation, ring type choice and leaflet management. Large-scale, prospective studies are warranted to assess the true effect of leaflet management on FMS development.

## Figures and Tables

**Table 1:** Patients’ baseline characteristics and preoperative data in the FMS and nFMS groups.

Variable	FMS (36)	nFMS (179)	*p*
Age (years)	63.04 ± 11.36	60.52 ± 12.43	0.263
Sex, male	22 (61.1)	114 (63.7)	0.770
BSA (m^2^)	1.67 ± 0.21	1.69 ± 0.20	0.478
Paroxysmal/persistent Af	11/7 (30.6/19.4)	41/46 (22.9/25.7)	0.545
DM	11 (30.6)	41 (22.9)	0.329
ESRD	4 (11.1)	7 (3.9)	0.091
CCr (mL/min)	69.52 ± 43.68	75.63 ± 36.95	0.399
LVEF (%)	59.91 ± 12.74	63.78 ± 13.02	0.107
LVEF < 40%	4 (11.1)	11 (6.1)	0.286
PASP (mmHg)	52.37 ± 17.76	48.21 ± 19.82	0.244
PASP ≥ 60 mmHg	13 (36.1)	52 (29.1)	0.401
Severe TR	3 (8.3)	19 (10.6)	>0.99
LVEDD (cm)	5.83 ± 1.14	5.87 ± 0.93	0.834
LVESD (cm)	3.88 ± 1.06	3.78 ± 0.88	0.583
LA diameter (cm)	4.51 ± 0.91	4.76 ± 1.04	0.184
NYHA Fc(I/II/III/IV)	1/11/22/2	8/79/85/7	0.417
MR pathology (Primary/Secondary)	27/9	153/26	0.121
Leaflet lesion (anterior/posterior/both/none)	11/10/5/10	32/85/34/28	0.046 *
Annulus dilatation	15 (41.7)	69 (38.5)	0.727
Segment involvement ^a^	1.22 ± 1.07	1.44 ± 1.03	0.238

Values are number (%) or mean ± standard deviation. ^a^ indicates the number of affected segments within the six mitral parts. * *p* < 0.05, significant difference. Abbreviations: Af, atrial fibrillation; BSA, body surface area; CCr, creatinine clearance rate; DM, diabetes mellitus; ESRD, end-stage renal disease; FMS, functional mitral stenosis; LA, left atrial; LVEF, left ventricular ejection fraction; LVEDD, left ventricular end-diastolic diameter; LVESD, left ventricular end-systolic diameter; MR, mitral regurgitation; nFMS, no functional mitral stenosis; NYHA Fc, New York Heart Association Classification functional class; PASP, pulmonary artery systolic pressure; TR, tricuspid regurgitation.

**Table 2:** Intraoperative characteristics of the FMS and nFMS groups.

Variable	FMS (36)	nFMS (179)	*p*
CABG	10 (27.8)	36 (20.1)	0.307
AVR	8 (22.2)	13 (7.3)	0.006 *
Maze	6 (16.7)	29 (16.2)	0.945
Aorta	3 (8.3)	4 (2.2)	0.093
TVP/TVR	15 (41.7)	74 (41.3)	0.971
Mitral ring size	27.83 ± 1.61	29.40 ± 2.52	0.0004 *
Mitral ring type (Memo 3D/Physio I/Physio II/IMR ring)	24/10/0/2	91/84/3/1	0.019 *
Leaflet management			
Resection only	2 (5.6)	39 (21.8)	0.021 *
Neochordae-only	20 (55.6)	51 (28.5)	0.002 *
Resection + neochordae	3 (8.3)	50 (27.9)	0.011 *
Edge-to-edge	2 (5.6)	1 (0.6)	0.073
Chordae transfer	1 (2.8)	9 (5.0)	>0.99
Patch repair	3 (8.3)	2 (1.1)	0.034 *

Values are number (%) or mean ± standard deviation. * *p* < 0.05, significant difference. Abbreviations: AVR, aortic valve replacement; CABG, coronary artery bypass grafting; FMS, functional mitral stenosis; nFMS, no functional mitral stenosis; TVP, tricuspid valve annuloplasty; TVR, tricuspid valve replacement.

**Table 3:** Primary and secondary outcomes.

Variable	FMS (36)	nFMS (179)	*p*
Surgical mortality	0 (0)	0 (0)	>0.99
Hospital mortality	0 (0)	1 (0.6)	>0.99
Post-MR > mild	8 (20.5)	18 (9.9)	0.041 *
Post-TR > mild	6 (20.5)	26 (14.4)	0.742
Post-LVEF (%)	61.27 ± 15.07	61.13 ± 12.80	0.953
Post-PASP (mmHg)	41.38 ± 20.96	30.60 ± 11.69	<0.0001 *
Post-LVEDD (cm)	4.97 ± 0.86	4.94 ± 0.71	0.830
Post-LVESD (cm)	3.28 ± 0.96	3.29 ± 0.79	0.929
Post LA diameter (cm)	4.27 ± 0.89	4.19 ± 1.01	0.684
Mean TMPG (mmHg)	7.27 ± 2.35	3.37 ± 1.01	<0.0001 *
Peak TMPG (mmHg)	15.92 ± 5.01	8.86 ± 2.99	<0.0001 *
MVA ^a^ (cm^2^)	2.18 ± 0.62	2.33 ± 0.70	0.262
Follow-up duration (month)	43.05 ± 18.74	43.58 ± 28.41	0.915
Echo follow-up duration (month)	34.33 ± 19.80	35.16 ± 27.31	0.862

Values are number (%) or mean ± standard deviation. ^a^ The mitral valve area was estimated by dividing 220 by the pressure half-time. * *p* < 0.05, significant difference. Abbreviations: FMS, functional mitral stenosis; LA, left atrial; LVEF, left ventricular ejection fraction; LVEDD, left ventricular end-diastolic diameter; LVESD, left ventricular end-systolic diameter; MR, mitral regurgitation; MVA, mitral valve area; nFMS, no functional mitral stenosis; PASP, pulmonary artery systolic pressure; TMPG, trans-mitral pressure gradient; TR, tricuspid regurgitation.

**Table 4:** Logistic regression for FMS predictors.

Variable	Coefficient	Std. Error	*p*	Coefficient	Std. Error	OR	95% CI	*p*	VIF
	Univariable			Multivariable					
ESRD	1.459	0.681	0.032 *	1.009	0.920	2.743	0.451–16.670	0.273	1.212
Leaflet lesion	Posterior = 1								1.665
No leaflet lesion	0.656	0.570	0.249						
Anterior	1.417	0.506	0.005 *	0.431	0.638	1.540	0.440–5.383	0.498	
Bi-leaflets	−0.441	0.811	0.586						
AVR	1.219	0.549	0.026 *	1.846	1.067	6.338	0.782–51.350	0.083	1.875
Aorta	1.549	0.793	0.051	0.722	1.185	2.059	0.201–21.015	0.542	1.403
Mitral ring size	−0.403	0.120	0.0008 *	−0.369	0.151	0.691	0.513–0.930	0.014 *	1.175
Mitral ring type	Memo 3D = 1								1.381
Physio I	−0.923	0.467	0.048 *	−1.123	0.778	0.325	0.070–1.495	0.149	
Physio II	−18.008	7152.429	0.998						
IMR ring	1.486	1.436	0.301						
Resection only	−2.051	1.035	0.047 *	−0.271	1.375	0.762	0.015–11.298	0.843	2.312
Neochordae-only	1.476	0.426	0.0005 *	1.303	0.880	3.681	0.655–20.684	0.138	2.567
Resection + neochordae	−2.409	1.033	0.019 *	−1.457	1.392	0.232	0.015–3.562	0.294	2.850
Edge-to-edge	21.794	9310.823	0.998						
Patch repair	1.810	1.022	0.076	4.531	1.707	92.905	3.271–2638.442	0.007 *	1.067

* *p* < 0.05, significant difference. Abbreviations: AVR, aortic valve replacement; CI, confidence interval; ESRD, end-stage renal disease; LVEF, left ventricular ejection fraction; OR, odds ratio; VIF, variance inflation factor.

**Table 5:** Baseline characteristics and preoperative data of patients with degenerative MR in the FMS and nFMS groups.

Variable	FMS (23)	nFMS (138)	*p*
Age (years)	66.03 ± 9.19	60.17 ± 12.98	0.039 *
Sex, male	15 (65.2)	86 (62.3)	0.790
BSA (m^2^)	1.67 ± 0.21	1.72 ± 0.20	0.294
Paroxysmal/persistent Af	9/7 (39.1/30.4)	29/41 (21.0/29.7)	0.119
DM	6 (26.1)	30 (21.7)	0.644
ESRD	1 (12.8)	2 (3.9)	0.372
CCr (mL/min)	68.82 ± 46.07	77.65 ± 37.20	0.311
LVEF (%)	64.00 ± 9.01	66.81 ± 9.28	0.178
LVEF < 40%	0 (0)	1 (0.7)	>0.99
PASP (mmHg)	55.45 ± 15.25	48.51 ± 19.43	0.104
PASP ≧ 60 mmHg	9 (39.1)	41 (29.7)	0.367
Severe TR	1 (4.3)	13 (9.4)	0.694
LVEDD (cm)	5.61 ± 0.98	5.85 ± 0.79	0.209
LVESD (cm)	3.60 ± 0.77	3.63 ± 0.71	0.850
LA diameter (cm)	4.73 ± 0.85	4.89 ± 1.04	0.527
NYHA Fc(I/II/III/IV)	1/10/12/0	5/67/64/2	0.894
Leaflet lesion (anterior/posterior/both)	10/8/2	27/75/25	0.046 *
Annulus dilatation	8 (34.8)	44 (31.9)	0.783
Segment involvement ^a^	1.30 ± 0.70	1.54 ± 0.96	0.254

Values are number (%) or mean ± standard deviation. ^a^ indicates the number of affected segments within the six mitral parts. * *p* < 0.05, significant difference. Abbreviations: Af, atrial fibrillation; BSA, body surface area; CCr, creatinine clearance rate; DM, diabetes mellitus; ESRD, end-stage renal disease; FMS, functional mitral stenosis; LA, left atrial; LVEF, left ventricular ejection fraction; LVEDD, left ventricular end-diastolic diameter; LVESD, left ventricular end-systolic diameter; MR, mitral regurgitation; nFMS, no functional mitral stenosis; NYHA Fc, New York Heart Association Classification functional class; PASP, pulmonary artery systolic pressure; TR, tricuspid regurgitation.

**Table 6:** Intraoperative characteristics of patients with degenerative MR in the FMS and nFMS groups.

Variable	FMS (23)	nFMS (138)	*p*
CABG	4 (17.4)	20 (14.5)	0.752
AVR	3 (13.3)	3 (2.2)	0.038 *
MAZE	6 (26.1)	23 (16.6)	0.277
Aorta	2 (8.7)	2 (1.4)	0.098
TVP/TVR	11 (47.8)	55 (39.9)	0.473
Mitral ring size	27.74 ± 1.51	29.59 ± 2.64	0.001 *
Mitral ring type (Memo 3D/Physio I/Physio II/IMR ring)	18/5/0/0	69/67/2/0	0.040 *
Leaflet management			
Resection only	2 (8.7)	37 (26.8)	0.068
Neochordae-only	19 (82.6)	42 (30.4)	<0.0001 *
Resection + neochordae	1 (4.3)	43 (31.2)	0.005 *
Edge-to-edge	2 (8.7)	0 (0)	0.019 *
Chordae transfer	1 (4.3)	8 (5.8)	>0.99
Patch repair	0 (0)	0 (0)	>0.99

Values are number (%) or mean ± standard deviation. * *p* < 0.05, significant difference. Abbreviations: AVR, aortic valve replacement; CABG, coronary artery bypass grafting; FMS, functional mitral stenosis; nFMS, no functional mitral stenosis; TVP, tricuspid valve annuloplasty; TVR, tricuspid valve replacement.

**Table 7:** Primary and secondary outcomes among patients with degenerative MR.

Variable	FMS (23)	nFMS (138)	*p*
Surgical mortality	0 (0)	0 (0)	>0.99
Hospital mortality	0 (0)	1 (0.6)	>0.99
Post-MR > mild	4 (17.4)	13 (9.4)	0.270
Post-TR > mild	2 (8.7)	17 (12.3)	>0.99
Post-LVEF (%)	62.47 ± 13.55	62.83 ± 11.14	0.889
Post-PASP (mmHg)	36.78 ± 15.39	29.62 ± 10.78	0.006 *
Post-LVEDD (cm)	4.76 ± 0.65	4.89 ± 0.69	0.390
Post-LVESD (cm)	3.05 ± 0.62	3.19 ± 0.66	0.335
Post LA diameter (cm)	4.22 ± 0.97	4.30 ± 1.02	0.717
Mean TMPG (mmHg)	7.18 ± 2.68	3.29 ± 0.98	<0.0001 *
Peak TMPG (mmHg)	15.75 ± 5.83	8.68 ± 2.97	<0.0001 *
MVA ^a^ (cm^2^)	2.26 ± 0.54	2.26 ± 0.71	0.963
Follow-up duration (month)	41.79 ± 18.71	46.08 ± 28.69	0.490
Echo follow-up duration (month)	32.35 ± 18.88	37.75 ± 27.28	0.363

Values are number (%) or mean ± standard deviation. ^a^ The mitral valve area was estimated by dividing 220 by the pressure half-time. * *p* < 0.05, significant difference. Abbreviations: FMS, functional mitral stenosis; LA, left atrial; LVEF, left ventricular ejection fraction; LVEDD, left ventricular end-diastolic diameter; LVESD, left ventricular end-systolic diameter; MR, mitral regurgitation; MVA, mitral valve area; nFMS, no functional mitral stenosis; PASP, pulmonary artery systolic pressure; TMPG, trans-mitral pressure gradient; TR, tricuspid regurgitation.

**Table 8:** Logistic regression analysis for predicting FMS in patients with degenerative MR.

Variable	Coefficient	Std. Error	*p*	Coefficient	Std. Error	OR	95% CI	*p*	VIF
	Univariable			Multivariable					
Age	0.043	0.023	0.063	0.008	0.030	1.008	0.949–1.070	0.792	1.104
Leaflet lesion	posterior = 1								1.125
Anterior	1.574	0.551	0.004 *	0.402	0.751	1.495	0.343–6.519	0.592	
Bi-leaflets	−0.774	1.096	0.480						
AVR	1.978	1.032	0.055	2.813	2.416	16.670	0.146–1899.354	0.244	2.306
Aorta	1.978	1.032	0.055	1.451	2.334	4.269	0.044–414.492	0.534	2.300
Mitral ring size	−0.401	0.141	0.004 *	−0.346	0.201	0.706	0.476–1.049	0.085	1.218
Mitral ring type	Memo 3D = 1								1.367
Physio I	−1.560	0.654	0.017 *	−0.737	1.131	0.478	0.052–4.392	0.514	
Physio II	−18.153	7564.073	0.998						
Resection only	−1.905	1.046	0.068	1.091	1.900	2.980	0.071–123.582	0.565	1.455
Neochordae-only	3.006	0.772	0.0001 *	3.682	1.512	39.752	2.049–771.051	0.014 *	1.435
Resection + neochordae	−21.057	7580.231	0.998						
Edge-to-edge	21.960	9283.290	0.998						

* *p* < 0.05, significant difference. Abbreviations: AVR, aortic valve replacement; CI, confidence interval; ESRD, end-stage renal disease; LVEF, left ventricular ejection fraction; OR, odds ratio; Preop, preoperative; VIF, variance inflation factor.

## Data Availability

Data is available on request from the authors.

## Supplementary Materials

The following supporting information can be downloaded at: https://www.mdpi.com/article/10.3390/jcdd10110470/s1, Supplementary Figure S1. Mitral annuloplasty ring size distribution between FMS and nFMS group. Supplementary Table S1. Reasons for patients who did not undergo follow-up echocardiography.
