# Supplementary material for: The Predictors and Outcomes of Functional Mitral Stenosis following Surgical Mitral Valve Repair: A Retrospective Analysis
Source: J Cardiovasc Dev Dis. 2023 Nov 19;10(11):470. doi: 10.3390/jcdd10110470 (PMC10672255; doi:10.3390/jcdd10110470)
Supplement: Supplementary file 1 [file jcdd-10-00470-s001.zip › Supplementary Table S1.pdf]

Supplementary Table S1. Reasons for patients who did not undergo follow-up echocardiography.

Reason	Patient number
Hospital mortality	4
Death occurring after discharge but before the first follow-up echocardiography	5
Follow-up echocardiography performed at another hospital due to transportation issues	3
Undergoing a heart transplant prior to the first follow-up echocardiography.	1
