# Supplementary figures and images for: The Predictors and Outcomes of Functional Mitral Stenosis following Surgical Mitral Valve Repair: A Retrospective Analysis
Source: J Cardiovasc Dev Dis. 2023 Nov 19;10(11):470. doi: 10.3390/jcdd10110470 (PMC10672255; doi:10.3390/jcdd10110470)

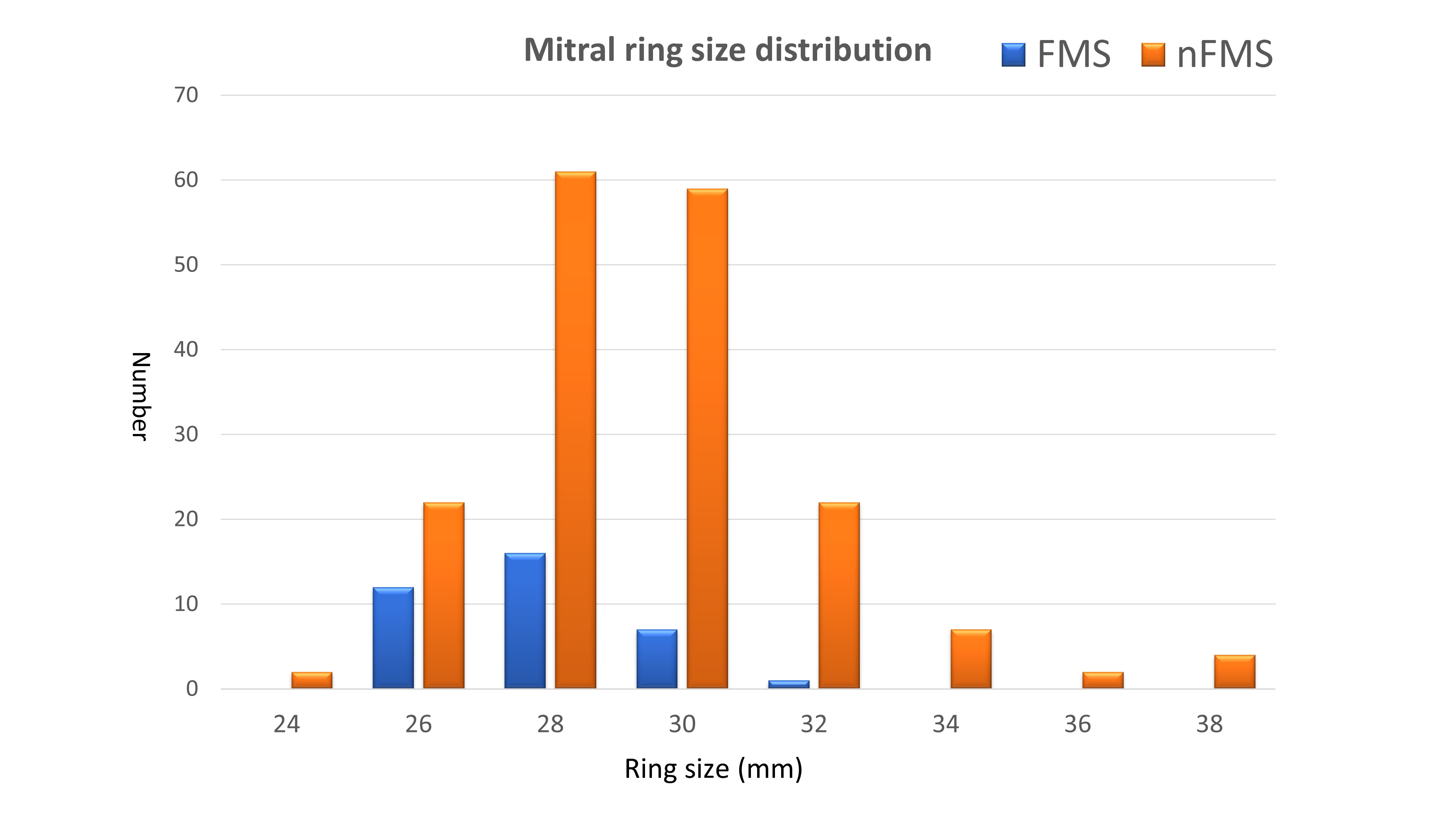

Supplement: Supplementary file 1 [file jcdd-10-00470-s001.zip › Supplementary Figure 1.jpg]
